# Quinine

**Published:** 1863-08

**Authors:** R. Walker


Quinine.—*Therapeutical Action.*—Quinine is held to be a direct tonic on insufficient grounds, its real action is that of a sedative to the efferent nerves of the sympathetic, for the following reasons: That it requires a very much larger dose of quinine to produce its physiological effects in robust persons than in the debilitated; and in extreme asthenia, even small doses are poisonous. That it decreases the force and frequency of the pulse. When given in inflammatory fever, it restores the activity of the secretions. The following practical points will be found true. Quinine is only useful in dyspepsia, which results from suppression of the digestive secretions from febrile irritation. It should be used rather in sthenic than asthenic cases of inflammation. It is not suitable in diseases of the heart.—(Mr, R.Walker,) *Med. Times and Gazette, Feb.* 21, 1863.

